# Improved adaptive radiotherapy to adjust for anatomical alterations during curative treatment for locally advanced lung cancer

**DOI:** 10.1016/j.phro.2021.04.003

**Published:** 2021-05-08

**Authors:** Maria Moksnes Bjaanæs, Erlend Peter Skaug Sande, Øyvind Loe, Christina Ramberg, Tove Mette Næss, Andreas Ottestad, Lotte V. Rogg, Jørund Graadal Svestad, Vilde Drageset Haakensen

**Affiliations:** a Dept of Oncology, Oslo University Hospital, Oslo, Norway; b Dept of Medical Physics, Oslo University Hospital, Oslo, Norway; c Dept of Cancer Genetics, Oslo University Hospital, Oslo, Norway

**Keywords:** Adaptive radiotherapy, Cone-beam CT, Re-calculation of radiation doses, Lung cancer

## Abstract

Anatomical changes during chemoradiation for lung cancer may decrease dose to the target or increase dose to organs at risk. To assess our ability to identify clinically significant anatomical alterations, we followed 67 lung cancer patients by daily cone-beam CT scans to ensure correct patient positioning and observe anatomical alterations. We also re-calculated the original dose distribution on a planned control CT scan obtained halfway during the treatment course to identify anatomical changes that potentially affected doses to the target or organs at risk. Of 66 patients who completed the treatment, 12 patients needed adaptation, two patients were adapted twice. We conclude that daily cone-beam CT and routines at the treatment machine discover relevant anatomical changes during curative radiotherapy for patients with lung cancer without additional imaging.

## Highlights

• Daily imaging during long-term fractionated radiotherapy is necessary to discover anatomical changes during treatment.
• A clearly defined adaptive strategy based on daily cone-beam CTs is sufficient to discover significant alterations.
• An adaptive strategy depends on consistent delineation.

## 1 Introduction

Lung cancer remains the leading cause of cancer-related deaths worldwide [1] and treatment of locally advanced disease is still a major challenge. Recent improvements in diagnostic imaging, chemoradiation and adjuvant immunotherapy have contributed to increased survival seen in this group; however, treatment is still associated with high risk of recurrence and possible lethal toxicities [2], [3], [4].

A major advance in radiotherapy over the last recent years has been the evolution of image-guided radiotherapy (IGRT). Use of daily pre-treatment cone beam computed tomography (CBCT) is especially useful as it may allow decreased setup margins [5], [6], [7], [8]. Decreased margins allow improved sparing of healthy tissue and may also increase the potential of dose escalation. Dose escalation is, however, highly debatable after the results from the RTOG 0617 trial [9].

Although reduction of margins to the treatment volume has clear advantages, it makes treatment plans more vulnerable to anatomical changes during the course of radiotherapy. Such changes could be related to weight loss, atelectasis, pleural effusions, baseline shifts in relative position between tumor and lymph nodes and alterations in tumor volume. Changes in tumor volume alone is found to be 30% in average after 50 Gy in 2-Gy fractions [10]. To account for these changes, adaptive radiotherapy (ART) has been developed. ART refers to modification of treatment plans based on systematic changes observed during the course of radiotherapy [11], [12] and several publications have described ART specifically for lung cancer patients [13], [14], [15], [16], [17], [18], [19], [20], [21].

When implementing ART, it is necessary to determine what kind of changes should trigger adaption. Møller et al [15] demonstrated the efficacy of an adaptive strategy for lung cancer patients with strict trigger criteria based on daily online evaluation of pre-treatment CBCTs.

The aim of the study was to control and improve our adaptive strategy for curative radiotherapy of lung cancer patients. To do that, we systematically evaluated anatomical alterations seen in the daily CBCT scans obtained at the treatment machine, using a pre-specified checklist. Furthermore, we performed control CT scans (cCT) used to re-calculate the dose distribution from the original treatment plans. Through this work we aimed to simplify the checklist used at the treatment machine to evaluate anatomical alterations. We also aimed to improve our adaptive strategy for this patient group. We here present our findings, the resulting adaptive strategy and illustrate some adaptive challenges when treating locally advanced lung cancer with curative radiotherapy.

## 2 Material and methods

All lung cancer patients admitted to our hospital for curative fractionated radiotherapy between May 2018 and January 2019, were asked to participate in the study. The regional ethical committee accepted this as an internal quality control project and the local ethical committee approved the project and publication of data. Oral accept of participation was given by 67 patients. A total of 67 patients were followed through curative radiotherapy. One patient stopped treatment after 7 fractions due to sepsis and ileus and is not included in this presentation. Patient characteristics are shown in Table A.2 (supplementary material). 53 patients were treated for non-small-cell lung cancer (NSCLC) and 14 for small-cell lung cancer (SCLC). Patients with SCLC were treated with 45 Gy in 1.5 Gy-fractions given twice daily. Most (70%) of patients with NSCLC received 66 Gy in 2-Gy fractions (range 60–70 Gy). 60 patients (90%) received concurrent chemotherapy. The median age was 66 (range 44–79).

All patients had a free breathing planning CT scan (pCT) for delineation and treatment planning, and a 4DCT scan for assessment of respiratory motion. The gross tumor volume (GTV) of the tumor and pathological lymph nodes was delineated on the free breathing CT and expanded by the motion observed on the 4DCT scan to create an internal GTV (iGTV). The clinical target volume (CTV) was defined by adding a 5 mm isotropic margin to the iGTV, and then cropped for bone and large vessels. For both primary tumor and lymph nodes the CTV was expanded by 5–8 mm to create the planning target volume (PTV), with margins dependent on treatment machine differences due to the institution’s practice. Treatment planning was performed in Varian Eclipse v13.6 or RayStation v5 using either volumetric-modulated arc therapy (VMAT) or intensity-modulated radiotherapy (IMRT).

Daily pre-treatment CBCTs were acquired, and images were matched to the primary tumor. Detailed instructions for CBCT image evaluation included a checklist of bone vs tumor match, the 50-Gy isodose related to the spinal canal, mismatch of GTV, changes in atelectasis, infiltrates, pleural effusions, heart, body surface or tumor diameter (Table A.1) Whenever a major anatomical change appeared or if a checklist question was answered ‘yes’ three days in a row, a physicist or oncologist was contacted. If deemed necessary, a cCT scan was obtained, and the original delineations were copied to the cCT and adjusted to the “new” anatomy if necessary. The dose distribution from the original treatment plan was re-calculated on this cCT to assess possible alterations of doses to target and organs at risk. ‘Major anatomical changes’ was a wording added to encourage radiation personnel to action if they observed alterations that were not covered by the checklist. In addition, all patients had a preplanned cCT midway during treatment that was co-registered with the pCT. Target volumes and OARs were re-delineated in the cCT. The original treatment plan dose distribution from the pCT was re-calculated on the cCT to evaluate target dose coverage and OAR doses. Re-planning was done when re-calculation on the cCT showed PTV D_95% vol_ below 95% of the prescribed dose, or when doses to an OAR delivered during the treatment period exceeded OAR-restrictions. Shrinking the CTV following tumor shrinkage in order to reduce toxicities, has been evaluated before [22]. We intended to keep the CTV constant despite reduction in GTV to avoid exclusion of microscopic disease. Reducing the CTV was performed if deemed necessary to allow completion of the treatment course with acceptable doses to OAR.

The checklist for CBCTs during radiotherapy would be updated to incorporate additional checkpoints if the study indicated that original list did not identify all clinically meaningful alterations. We would also aim at simplifying the list if possible.

## 3 Results

Twelve patients needed adaptation of the original treatment plan (18%) (Table 1). For eight patients, a CT scan was performed *prior to* the planned cCT, five of these were re-planned. The three who were not replanned had pleural effusion (one patient) or tumor shrink (two patients) that did not lead to violations of dose requirements. Six patients were re-planned at the time of the scheduled cCT. Three patient needed adaptation of the treatment plan after the planned cCT. Two patients needed adaptation twice during the treatment.

**Table 1:** Overview of 12 patients that received a total of 14 new treatment plans. The main reason for adaptation is indicated. *Target shift* relates to checklist point 3. *Pleural fluid* and *infiltration* relate to checklist point 5. *Target increase* or *decrease* relates to checklist point 7. cCT: Preplanned control CT. UC: Undifferentiated carcinoma, AC: Adenocarcinoma, SCC: Squamous cell carcinoma, SCLC: Small-cell lung cancer.

Patient	Before cCT	At cCT	After cCT	Total	Histology
1	Target shrink			1	UC
2		Target shrink		1	AC
3		↑Pleural fluid	↓Pleural fluid	2	AC
4			Infiltration	1	SCC
5			Target increase	1	SCC
6	Target increase			1	SCLC
7		Target increase		1	SCC
8	Target shrink			1	SCC
9		Target shift		1	AC
10	↑Infiltration	↓Infiltration		2	AC
11	Target increase			1	SCC
12		Target shrink		1	SCC
Total replanned	5	6	3	14	
Target shrink	2	2	0	4	
Target increase	2	1	1	4	
Target shift	0	1	0	1	
Infiltration	1	1	1	3	
Pleural fluid	0	1	1	2	

One of the six patients that received a new treatment plan after the scheduled cCT would not have been re-planned without the cCT as none of the checkpoints were identified at the treatment machine (patient 2 in Table 1). The patient was re-planned due to an increase in doses to the spinal canal (max 53.7 Gy). Re-examination of the images and calculated doses revealed an inconsistency of delineation responsible for the violation of dose constraints in one small area and with a consistent delineation, re-planning would not have been necessary (for detailed description and images, see patient 2 in the Appendix). The remaining violations leading to re-planning would all have been discovered on the daily CBCTs given the checklist used (Table A.1). All violations identified at the treatment machines were based on three of seven criteria in the checklist (tumor volume match, atelectasis/infiltrate/pleural effusion and changes in tumor diameter). The checklist was simplified as a result of the study and incorporated in our final adaptive strategy (Table 2). We removed the criteria concerning differences between bone match and online match and concerning lymph nodes match to delineated surrogates because clinically relevant alterations were detected by the remaining checkpoints. The criterium concerning tumor volume was simplified to “target volume seen outside CTV” since this is easier to identify for the treatment personnel.

**Table 2:** Our adaptive radiotherapy guidelines adjusted after the patient case series. Point 2 in this list contains the modified version of the checklist presented in Table A.1.

1) All treatment plans are presented and discussed during chart round the first week of treatment
2) Radiation personnel register for every treatment:
- Target volumes seen outside CTV
- 50 Gy isodose overlapping the spinal canal
- Larger anatomical alterations (pleural effusion, pneumonia, body contour, heart etc)
- Tumor diameter reduced by >10 mm or increased by >5 mm
3) A new CT for re-calculation of doses should be obtained if violations of matching-criteria (checklist) occur in three consecutive days
4) Immediate discussion/re-planning if target volume outside PTV
5) Adaptation is required if doses compared to original planning CT show:
- reduction in CTV D98% of >2 percentage points
- increased of global maximal dose outside PTV of >3 percentage points
- dose to OAR exceeding accepted dose

## 4 Discussion

Of 67 patients included, 66 completed curative radiotherapy, of which 12 needed adaptation of the treatment plan once or twice. All clinically significant alterations could be identified by use of our checklist. Alterations leading to adaptation included changes in tumor volume, pulmonary infiltration or effusion and a shift of tumor localization without changes in tumor volume. Our adaptive strategy was adjusted based on the results.

A knowledge-based adaptive strategy is mandatory for fractionated radiotherapy of lung cancer patients. Not all anatomical changes need adaptation. While anatomical alterations have been observed in 72%-83% of patients [23], [24], the fraction needing adaptation of the treatment plan has been reported lower (27% in [15], 48% in [14] and 60% in [24]). We found a need for adaptation in 18% of patients. Møller and colleagues showed that only minor alterations in adaptation-criteria could influence the rate of adaptations greatly [14]. Like previous studies [15], [24], changes in tumor volume was the main reason for adaptation. In our study, the majority (94%) of changes leading to adaptation were discovered before fraction 16 in line with Møller et al although they had a slightly higher fraction (29%) of adaptations from fraction 16 and later. Differences in the heart volume during chemoradiation of esophageal cancer of up to 6% has been reported [25], but we did not observe any instance in which the heart deviated a 1 cm from the original delineation or more.

Based on the checklist followed at the treatment machine (Table A.1), five out of six patients identified at the cCT had alterations that would trigger a cCT based on the checklist. The one who would not be identified from a CBCT with checklist, was selected for adaptation due to inconsistent delineation rather than anatomical changes. We therefore conclude that daily evaluations by CBCT with a checklist detect significant anatomical alterations and that routine cCTs and re-calculations are not necessary. We underline the importance of consistent delineation which is previously pointed out as the weakest point in the process of accurate radiotherapy [26]. Future imaging and delineation techniques, including automated delineation based on artificial intelligence, may greatly improve the delivery of radiotherapy [27]. Until these techniques are in place, contouring workshops internationally and locally, may help streamline inter-observer delineation.

Alterations of pulmonary infiltrations due to pneumonitis demanded adaptation twice in the same patient. Appearance or resolution of infiltrations may cause dosimetric changes without a shift of target volumes or OARs. We have therefore chosen to describe this patient in detail in the supplementary material (patient 1).

The Supplementary material describes two additional cases illustrating challenges in adaptive radiotherapy; consistent delineation of organs at risk (patient 2) and dosimetric effect of alterations in body outline (patient 3). Previous studies have found that outline variations have large dosimetric impact for radiation of cervical cancer and head and neck cancer, partly related to weight loss [28], [29]. We therefore underline the importance of supportive treatment to maintain stable weight and of re-calculating doses to target and risk organs if there are visible changes with uncertain clinical effect.

We adjusted our adaptive strategy based on our results by simplifying the checklist at the treatment machines and by specifying adaptation requirements. In addition, every patient is discussed at onset of treatment during weekly multidisciplinary meetings to identify less robust plans that will be discussed weekly. Examples of such plans include plans where doses to OAR are close to OAR-restrictions, plans with certain field arrangements vulnerable to changes in the radiation path or plans involving large tumors with peripheral atelectasis which may resolve during treatment. Adaptation is required if doses show reduction in CTV D98% of >2 percentage points compared to the original pCT, increase of global maximal dose outside PTV of >3 percentage points or dose to OAR exceeding accepted dose (see Table A.3).

Advances in imaging possibilities at the treatment machine allow more accurate knowledge of anatomical alterations occurring during treatment [27]. Since studies used to develop today’s radiation strategies did not always include CBCT and adaptation during treatment, the clinical consequence of adaptation is uncertain. Future studies should focus on clinical consequences of adaptation relating target doses to toxicity and survival. Improved adaptive strategies may also open the possibility for dose escalation without intolerable toxicities and hence reduce the risk of locoregional relapse and thereby improve survival outcomes for this group of patients.

Radiotherapy is constantly changing, implementing new technical advances into clinical practice. The increasing use of proton therapy poses particular challenges to adaptation especially when treating lung tumors due to large variations in tissue density and respiratory motion. Protons’ finite range and sharp dose fall-off makes treatment particularly sensitive to setup and range uncertainties, as well as changes in patient anatomy. Identifying patients susceptible for anatomical alterations is important when considering proton therapy as a treatment option.

We conclude that an adaptive strategy with daily CBCT and defined criteria that trigger new pCT and re-calculation, is sufficient to detect significant anatomical alterations occurring during curative radiotherapy of lung cancer patients. The results of this study have simplified our checklist and adapted our strategy accordingly.

## Funding

No funding was received for the current project.

Research data are stored in an institutional repository and will be shared upon request to the corresponding author.

## Declaration of Competing Interest

The authors declare that they have no known competing financial interests or personal relationships that could have appeared to influence the work reported in this paper.
