# Supplementary material for: Improved adaptive radiotherapy to adjust for anatomical alterations during curative treatment for locally advanced lung cancer
Source: Phys Imaging Radiat Oncol. 2021 May 8;18:51–4. doi: 10.1016/j.phro.2021.04.003 (PMC8254190; doi:10.1016/j.phro.2021.04.003)

Supplementary material

**Table A.1** Checklist for CBCT evaluation at the treatment machine. Each point would be evaluated daily. Alterations exceeding any checkpoint for three days in a row would elicit evaluation by a physicist and/or physician.

## Table A.1

| 1. Differences between bone match and online tumor match > 5 mm |
| --- |
| 2. 50 Gy isodose contour overlaps with the spinal canal |
| 3. Deviation of the lymph nodes or delineated surrogate structures > 5 mm |
| 4. Deviation of the delineated tumor GTV compared with online tumor match > 5 mm |
| 5. *Acquired or dissolving* atelectasis, infiltrate or pleural effusion |
| 6. Deviation of the heart (> 1 cm) or body surface (> 1,5 cm) |
| 7. Changes of the tumor diameter of > 5mm |

**Table A.2** Patient characteristics

| **Patient/tumor characteristic** |  | **N (%)** |
| --- | --- | --- |
| **Histology** | Small cell | 14 (21) |
|  | Non-small cell | 53 (79) |
|  | *Adenocarcinoma* | *25 (47)* |
|  | *Squamous cell carcinoma* | *26 (49)* |
|  | *Low differentiated carcinoma* | *1 (2)* |
|  | *Large cell Neuroendocrine* | *1 (2)* |
| **Gender** | Female | 31 (46) |
|  | Male | 36 (54) |
| **Stage** | Recurrent disease | 5 (7) |
|  | Adjuvant to surgery | 1 (1.5) |
|  | 2a | 1 (1.5) |
|  | 2b | 6 (9) |
|  | 3a | 30 (45) |
|  | 3b | 21 (31) |
|  | 3c | 1 (1.5) |
|  | 4 | 2 (3) |
| **Side** | Right | 36 (54) |
|  | Left | 27 (40) |
|  | Mediastinum | 4 (6) |
| **Smoking status** | Current smoker | 20 (30) |
|  | Former smoker | 43 (64) |
|  | Never smoker | 2 (3) |
|  | Missing data | 2 (3) |
| **ECOG status** | 0 | 24 (36) |
|  | 1 | 33 (50) |
|  | 2 | 10 (15) |
| **Dose and fractions** | 2 Gy x 33 | 47 (70) |
|  | 2 Gy x 30 | 4 (6) |
|  | 2 Gy x 35 | 2 (3) |
|  | 1.5 Gy x 2 x 15 | 14 (21) |
| **Concurrent** | Yes | 60 (90) |
| **chemotherapy** | No | 7 (10) |
| **Side-effects during** | Pneumonitis | 2 (3) |
| **treatment** | Esophagitis | 37 (55) |
| **Age** | Median | 66 |
|  | Range | 44 - 79 |

## Detailed patient descriptions

**Pulmonary infiltration**

**Patient 1** was treated for a NSCLC (T2aN3M0). Planning CT acquired ten days prior to start of radiotherapy revealed signs of pneumonitis in the patient’s right lung (Figure 1A). Build-up of pleural effusion and a massive increase in the pulmonary infiltration was observed on early CBCTs, making it difficult to distinguish tumor and inflammation. A second planning CT was acquired at fraction five (Figure 1B), and co-registered with the first planning CT. After re-delineation, the original treatment plan was recalculated on the new CT. The original CTV for the primary tumor was copied onto the new plan and was slightly increased despite tumor shrinkage.

Upon re-calculation of doses on the new CT, dose coverage of target volumes decreased to CTV mean dose for lymph nodes of 65.3 Gy to 65.8 Gy and for the primary tumor 64.4 Gy (66 Gy prescribed dose) (Figure 1C-D). Given the location of the primary tumor adjacent to the lung infiltration, greater changes could be expected. A plan with two full arcs resulted in small parts of the beams passing through the areas with density changes. Dose to OAR did not change significantly. Dose coverage of the primary tumor from the original treatment plan calculated on the first and second control CT are shown in Figure 1C-D. Although less dosimetric changes than might be expected, dose coverage was not optimal and the patient was re-planned with a new plan made with two semi arcs.

One week later, CBCT revealed considerable decrease in pneumonitis and pleural fluid. A third cCT was acquired (Figure 1E). Re-calculation of doses showed that the loss of lung consolidation increased doses to all target volumes and OARs (Figure 1 F-G) and CTV mean dose of lymph nodes ranged from 67.5 Gy to 70.5 Gy while mean dose to CTV tumor was 69.4 Gy. Changes in mean dose to selected OARs were: spinal canal: 23.7 Gy to 26.0 Gy, esophagus: 37.9 Gy to 44.7 Gy and left main bronchus: 57.8 Gy to 65.9 Gy. DVHs of these OARs are shown in Figure A.1). The increased dose to esophagus is considered significant. The use of two semi arcs versus two full arcs probably increased the dose changes seen due to larger parts of the beams passing through the patient in areas with large density changes.

**Figure A.1** A) A pulmonary infiltration was visible at the initial planning CT. B) At fraction 5, a new planning CT was performed due to increased pulmonary infiltration making it difficult to distinguish the primary tumor from the infiltration. Pleural effusion had appeared (arrow). The increased infiltration reduced dose to the primary tumor (D) compared with the dose in the initial plan (C). E) At fraction 13, a new planning CT was obtained due to reduction in pulmonary infiltration and pleural effusion. Dose for the target volume increased when the pulmonary infiltration disappeared (from F to G).

##
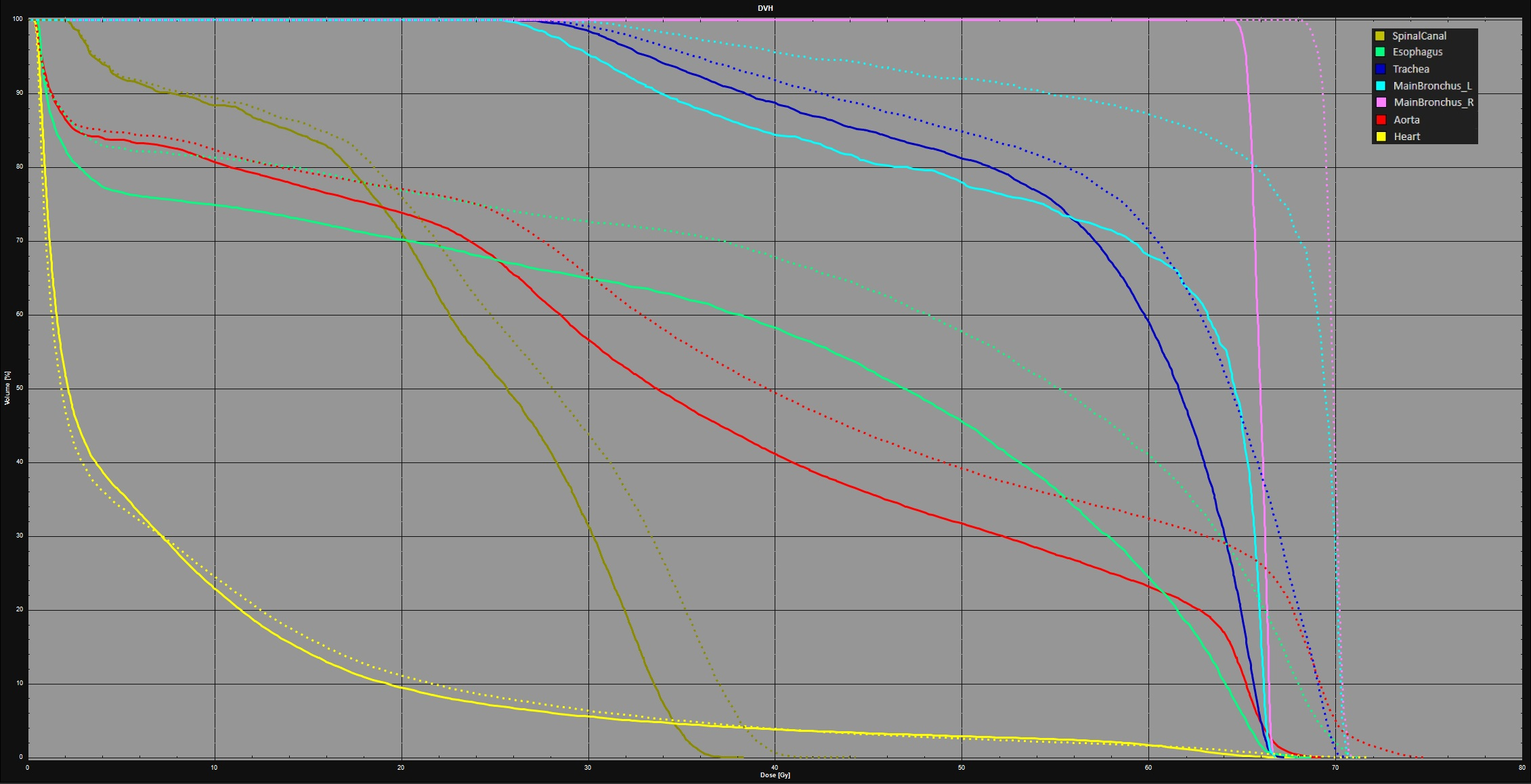
Figure A.1


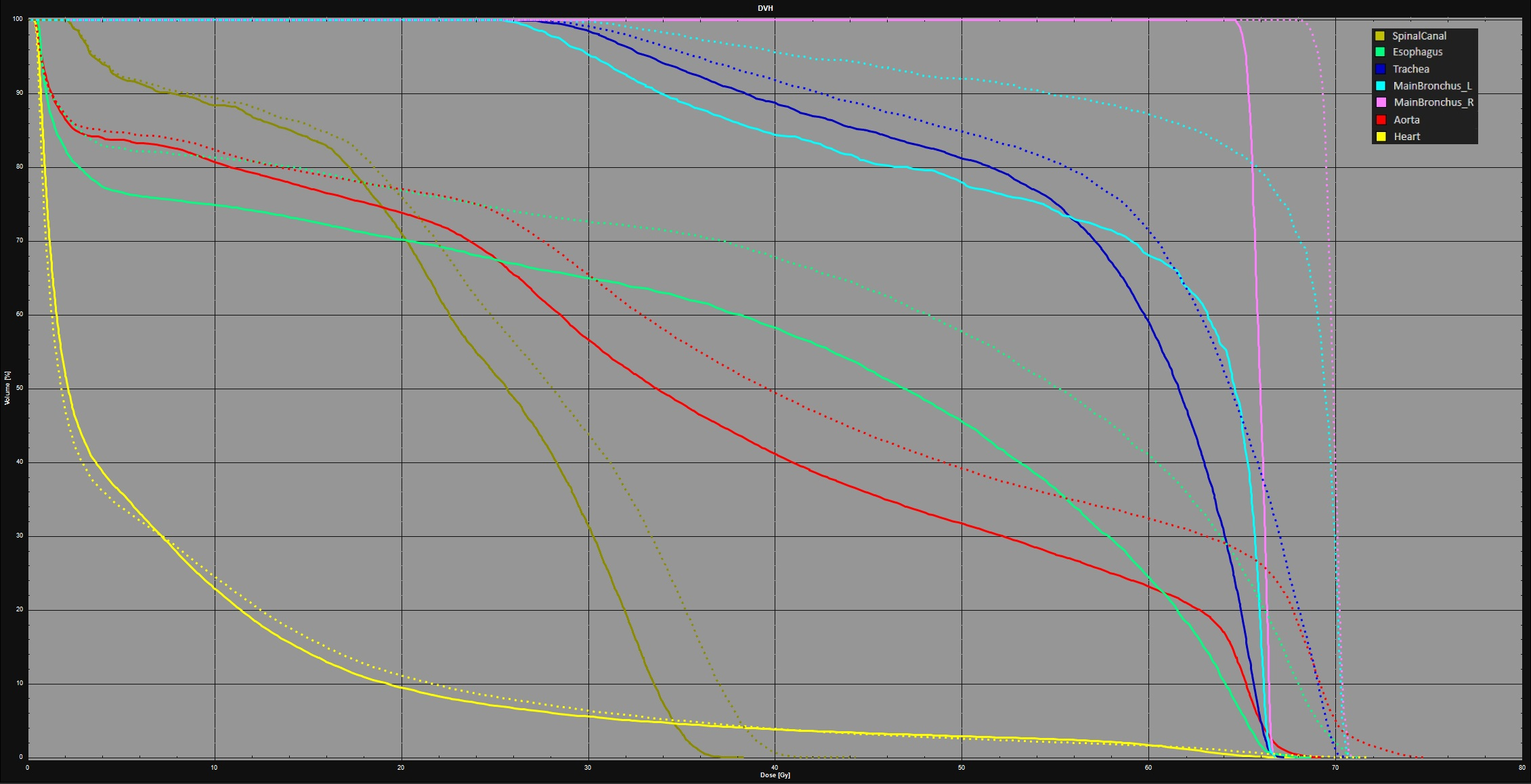


**Figure A.1**

Patient 1. DVHs in the second (solid line) and third (stippled) CTs for various
organs at risk. The doses invariably increased as a result of reduced
consolidations in the lung.

**Change in doses to the spinal canal; delineation**

**Patient 2** was treated for an adenocarcinoma in the right upper lobe cT2aN2M0. The maximum dose to the spinal canal in the original treatment plan was 49.7 Gy (Figure A.2 A). At fraction 14, the shrinkage was measured to 4 mm from the originally delineated GTV to the observed tumor border as seen on CBCT. A cCT the same day confirmed the magnitude of tumor shrinkage (Figure A.2 B). When re-calculating the original plan on the cCT, a maximum dose of 53.7 Gy to the spinal canal was observed – violating our dose constraint. A new treatment plan, in which all OAR constraints were met, was prepared.

The increased dose to the spinal canal was presumed to be caused by the observed tumor shrinkage, and thereby loss of dense tissue dose in front of the spinal canal. At closer look, the area of the spinal canal receiving too high radiation dose did not coincide with the area of tumor shrinkage. The increased dose was in fact caused by subtle differences in spinal canal delineation on the cCT compared to the original planning CT (Figure A.2 C). With a more consistent delineation on the cCT compared to the original planning CT, the re-calculation showed a lower maximum dose (51.5 Gy) to the spinal canal. This dose might have been deemed more acceptable, given that the patient was half-way through the course of radiation treatment, illustrating the importance of accurate delineation of OARs.
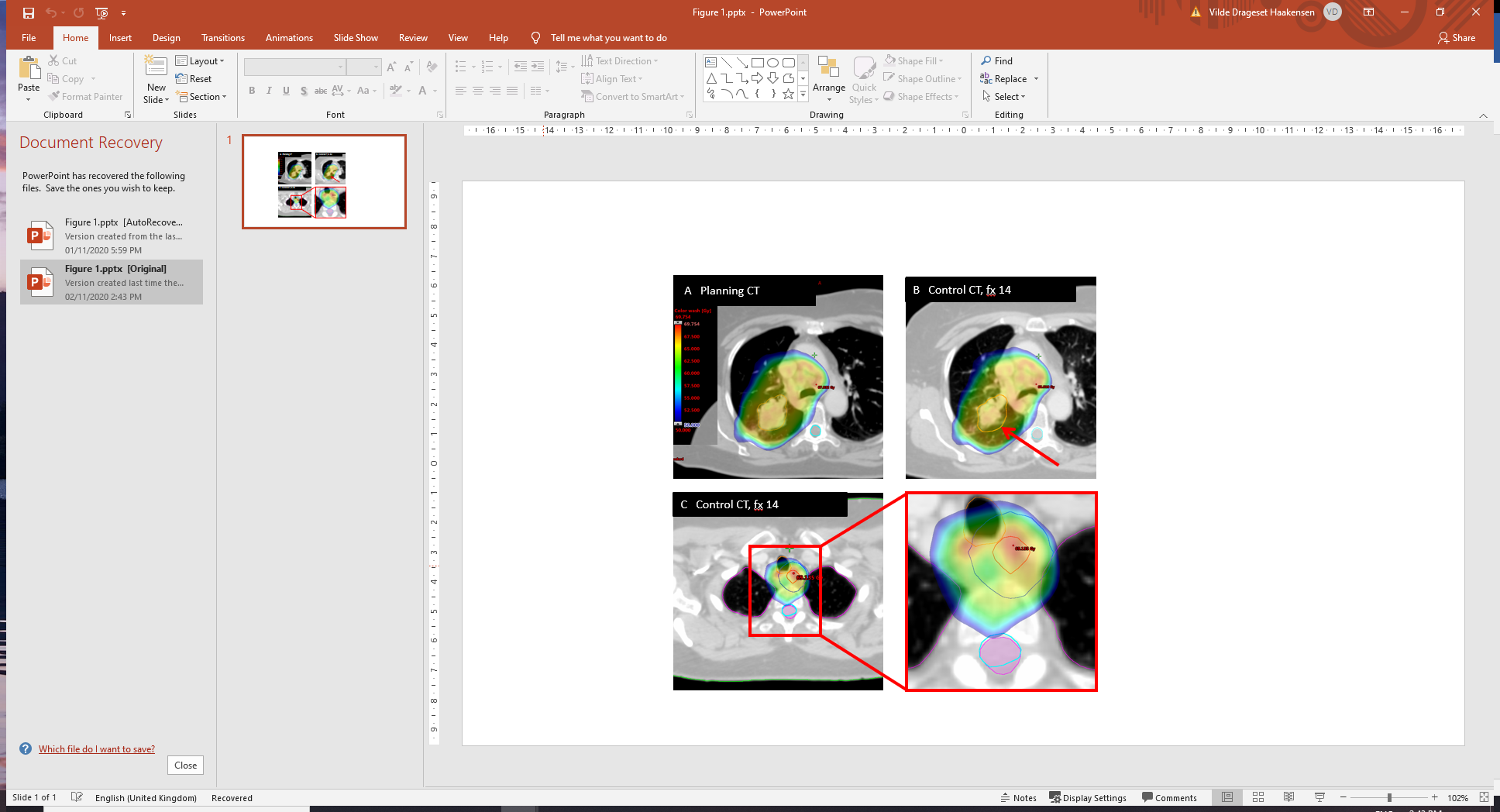


**Figure A.2.** A) On the treatment plan, the maximum dose to the spinal canal was 49.7 Gy. B) At fraction 14, the control CT showed a tumor shrinkage of 4 mm. Re-calculating the original plan on the control CT, resulted in a maximum dose of 53.7 Gy to the spinal canal. C) The area of the spinal canal that had increased doses on the control CT was found cranial to the tumor. On the control CT, the delineation of the spinal canal was inaccurate (turquoise). Upon a more correct delineation of the spinal canal (magenta), the re-calculation of the original plan showed a lower maximum dose (51.5 Gy).

**Changes in body contour**

**Patient 3** underwent chemoradiotherapy for a NSCLC with the primary tumor in the left upper lobe and mediastinal lymph nodes. A poor match for the lymph node target was observed at an early CBCT when matching on the primary tumor (Figure A.3 A). However, both targets were within their CTV and the mismatch was deemed not clinically significant. A slight discrepancy between the delineated body contour and the actual body contour was observed (Figure A.3 B). A systematic shift of 2-12 mm, with daily variations, was deemed not clinically important.

The increasing lymph node target misalignment complicated the daily CBCT match. The original treatment plan was recalculated on the cCT at fraction 17, indicating a poor dose coverage (Figure A.3 C) compared to the original plan (Figure A.3 D). The primary tumor was also under-dosed (Figure A.3 E-F). This was not expected, as the re-scanning was aligned tumor-to-tumor with the planning CT before recalculating the original plan. Tumor and lymph node CTVs had mean doses of 64.2 Gy and 64.3 Gy, respectively, in contrast to the prescribed 66 Gy. Dose-volume histograms (DVHs) for CTV and PTV union volumes are shown in Figure A.4**.**

The slight shift in body outline had a larger impact on delivered dose than expected. The actual body outline, as seen on the CBCT, was superimposed to the original planning CT (Figure A.5) to simulate the impact of the systematic shift – without taking into account the impact of the misaligned lymph node target. The original plan was recalculated on this modified, original CT scan. The resulting dose distribution showed very similar dose reduction of the primary tumor as found when recalculating the original plan on the cCT scan. The patient was treated with a new plan from fraction 18. The misaligned lymph node target volume might have been caused by changed respiration pattern at CT scanning compared to treatment, or effects of the treatment itself. The shifted body outline might have been caused by poor immobilization at CT scanning, or a non-representative body outline on the planning CT scan due to the patient being tense or anxious. To summarize, we expected the poor dose coverage to be caused mainly by poor alignment of the lymph node target compared to the planning CT. However, the changes in body contour turned out to impact the dose distribution more than expected.


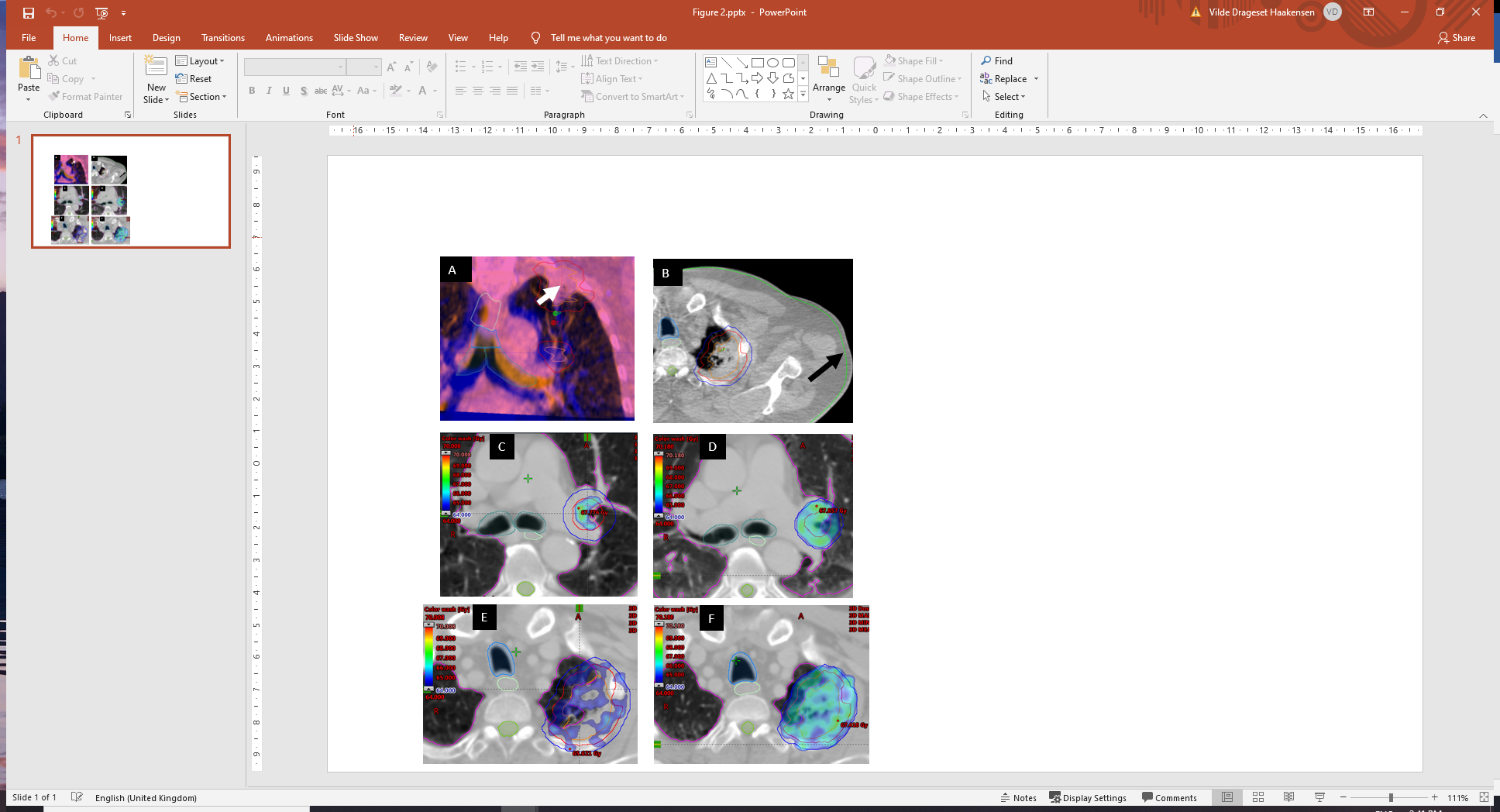


**Figure A.3** A) Matching on the primary tumor, a mismatch of CBCT (yellow) and planning CT (blue) in the area of the lymph nodes (arrow pointing at the lymph node CTV) was evident from early on in the treatment. A change in the body contour was also observed (arrow) (B). Re-calculation on a control CT at fraction 17 showed that the lymph node target volume had poor dose coverage (C) compared to the original treatment plan (D). Color wash indicates the 97% dose level. Dose coverage of the tumor is shown in (E) (control CT) and (F) (planning CT)

##
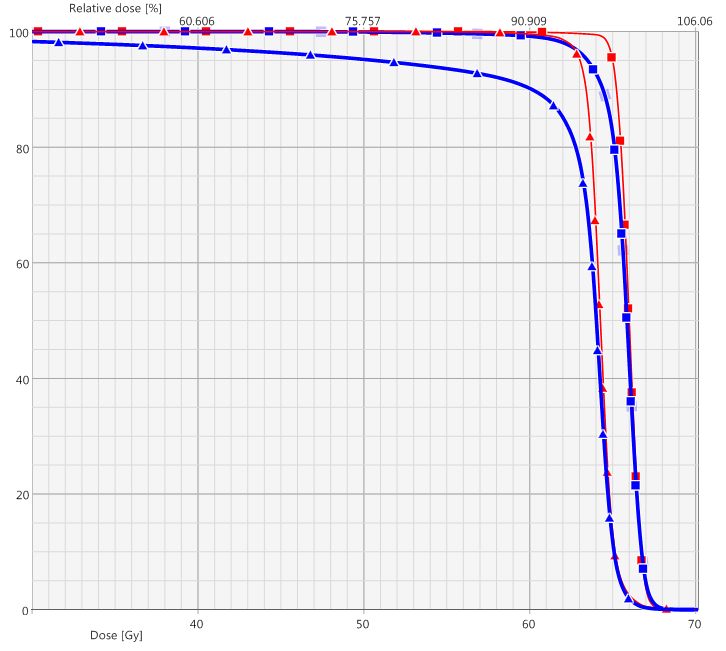
Figure A.4


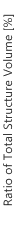


**Figure A.4** Patient 3. This figure demonstrates the altered dose-volume histograms (DVHs) for CTV (red) and PTV (blue) union volumes when the original plan was recalculated on the cCT. Tumor and lymph node CTVs had mean doses of 64.2 Gy and 64.3 Gy, respectively, in contrast to the prescribed 66 Gy. Tumor and lymph node PTVs had mean doses of 63.9 Gy and 57.8 Gy, respectively (planned to 65.6 Gy and 65.7 Gy).

## Figure A.5

**Figure A.5**

Patient 3. The actual body outline, as seen on the CBCT, was superimposed to the original planning CT to simulate the impact of the systematic shift (pink). This slight shift in body outline turned out to have a larger impact on delivered dose than expected.


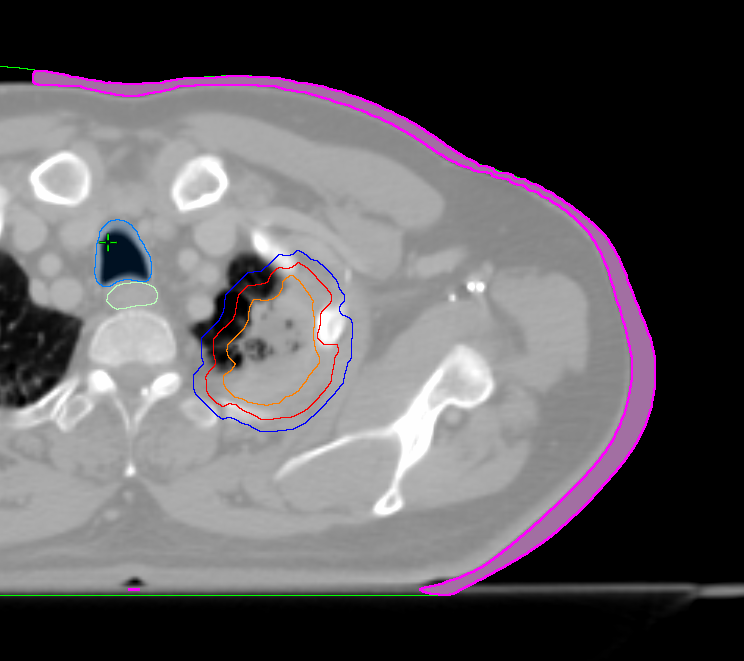

Supplement: Supplementary data 1 [file mmc1.docx]
